# Progress towards a comprehensive approach to maternal and neonatal immunization in the Americas

**DOI:** 10.26633/RPSP.2017.159

**Published:** 2017-12-20

**Authors:** Alba Maria Ropero Alvarez, Barbara Jauregui, Nathalie El Omeiri

**Affiliations:** 1 Pan American Health Organization Pan American Health Organization Washington, DC United States of America Pan American Health Organization, Washington, DC, United States of America.

**Keywords:** Immunity, maternally-acquired, prenatal care, immunization programs, Americas, Inmunidad materno-adquirida, atención prenatal, programas de inmunización, Américas, Imunidade materno-adquirida, cuidado pré-natal, programas de imunização, Américas

## Abstract

Maternal and neonatal immunization (MNI) is a core component of the new immunization model in the Americas, which transitioned from immunization of children to that of the entire family. Immunization during pregnancy protects the mother and the fetus by providing the neonate with maternal antibodies against disease. It has the potential to impact early childhood morbidity and mortality, and thus MNI has gained visibility and priority on the global health agenda.

The Region of the Americas is a leader in MNI, as seen by its elimination of congenital rubella syndrome in 2015 and the progress made toward neonatal tetanus elimination. In the Americas, 31 countries currently target pregnant women for influenza vaccination; and 21 countries—over 90% of the Region’s birth cohort—have nationwide newborn hepatitis B vaccination.

This paper describes the status of MNI in the Americas and identifies gaps in the evidence, obstacles to optimal implementation, and opportunities for future improvements. Catalysts for MNI in the Region have been political commitment, endorsement by scientific societies, an established “culture of vaccination,” widespread access to antenatal care, and context-specific communications; however, universal and equitable access for pregnant women and their newborns continues to be a formidable challenge, and additional vaccine safety and effectiveness evidence is needed. Continued efforts to integrate MNI with maternal and child health services will be critical to furthering the MNI platform as well.

## RESUMEN

La inmunización materna y neonatal es un componente central del nuevo modelo de inmunización en la Región de las Américas, que pasó de la inmunización infantil a la de toda la familia. La inmunización durante el embarazo protege a la madre y el feto dando al recién nacido los anticuerpos maternos contra las enfermedades. Tiene el potencial de repercutir en la morbilidad y la mortalidad en la primera infancia, por lo que la inmunización materna y neonatal ha adquirido visibilidad y prioridad en la agenda mundial de salud.

La Región de las Américas ocupa una posición de liderazgo en materia de inmunización materna y neonatal, como lo demuestra la eliminación del síndrome de rubéola congénita en el 2015 y los avances logrados para la eliminación del tétanos neonatal. Actualmente en 31 países de la Región se da prioridad a las embarazadas para que reciban vacunación antigripal y en 21 países —más de 90% de la cohorte de nacimiento de la Región— se incluye la vacunación de los recién nacidos contra la hepatitis B a nivel nacional.

En este documento se describe la situación de la inmunización materna y neonatal en la Región de las Américas y se señalan las lagunas en la evidencia, los obstáculos a la implementación óptima y las oportunidades para las mejoras futuras. Los catalizadores de la inmunización materna y neonatal en la Región han sido el compromiso político, el aval de las sociedades científicas, una “cultura de vacunación” establecida, el acceso generalizado a la atención prenatal y las comunicaciones específicas para cada contexto; sin embargo, el acceso universal y equitativo de las embarazadas y los recién nacidos sigue siendo un reto enorme y se necesitan más datos científicos sobre la seguridad y efectividad de las vacunas. La continuación de los esfuerzos para integrar la inmunización materna y neonatal en los servicios de salud maternoinfantil será fundamental para promover también la plataforma a favor de esta inmunización.

## RESUMO

A imunização materna e neonatal é peça fundamental do novo modelo de imunização nas Américas, com a transição da vacinação de crianças à vacinação de toda a família. A vacinação da gestante protege a mãe e o feto ao proporcionar ao recém-nascido anticorpos maternos contra doenças. A imunização materna e neonatal possivelmente repercute na primeira infância reduzindo a morbidade e a mortalidade e, portanto, ganhou visibilidade sendo considerada prioritária na agenda global de saúde.

A Região das Américas é líder em imunização materna e neonatal, tendo alcançado a eliminação da síndrome da rubéola congênita em 2015 e avançado para a eliminação do tétano neonatal. Existem atualmente programas de vacinação contra influenza para gestantes em 31 países e programas nacionais de vacinação contra hepatite B para recém-nascidos em 21 países (com uma cobertura superior a 90% da coorte de nascidos vivos na Região).

Este artigo apresenta um panorama da imunização materna e neonatal nas Américas, destaca as lacunas nas evidências científicas e os obstáculos à implementação ideal dos programas de vacinação e aponta oportunidades futuras para melhorias. Entre os fatores responsáveis pelo incentivo à imunização materna e neonatal na Região estão o compromisso político, o endosso das sociedades científicas, uma “cultura de vacinação” estabelecida, o amplo acesso à assistência pré-natal e a comunicação contextualizada. Porém, o acesso universal e equitativo das gestantes e seus recémnascidos à vacinação é ainda um grande desafio e se fazem necessárias mais evidências sobre a segurança e a efetividade das vacinas. Além disso, é imprescindível o empenho contínuo para integrar a imunização materna e neonatal aos serviços de saúde materno-infantil e seguir promovendo a plataforma de imunização materna e neonatal.

## RATONALE FOR MATERNAL AND NEONATAL IMMUNIZATION

Each year, approximately 290 000 women worldwide die from complications of pregnancy and childbirth (1). Currently, only one-half of pregnant women in developing countries receive the four prenatal checkups recommended by the World Health Organization (WHO; 2, 3). Regarding the Millennium Development Goals (MDGs), Latin America and the Caribbean did not meet the target “reduce maternal mortality by three-fourths by 2015,” which was set by the 5th goal, “Improve Maternal Health” (4). Additionally, preventable diseases continue to be the main cause of deaths among children less than 5 years of age; while neonatal mortality accounts for almost 40% of the estimated 6.6 million deaths in this age group and nearly 60% of all infant deaths (5).

Maternal immunization refers to immunization prior to pregnancy, during pregnancy, and in the post-partum period. MNI is a core component of the new immunization model, which in the Americas, transitioned from immunizing children to immunizing the whole family (6). Immunization during pregnancy protects not only the mother, but also the fetus through high concentrations of protective antibodies that move transplacentally. This transfer provides the neonate with maternal antibodies that protect against disease until active immunization of the infant can be accomplished. As such, MNI has the potential to impact early childhood morbidity and mortality. Infections such as influenza, tetanus, and pertussis are associated with adverse outcomes in young infants, i.e., prior to commencement or completion of the primary infant immunization series. Vaccination of newborns constitutes an important component of the maternal and neonatal immunization platform and includes both hepatitis B and Bacillus Calmette-Guérin (BCG) vaccines, optimally within the first 24 hours of life. Indeed, globally, two-thirds of hepatitis B-related deaths result from infection in the perinatal and early childhood period. This underscores infant hepatitis B immunization, beginning at birth, as the cornerstone of a hepatitis B prevention strategy (7).

In May 2012, the World Health Assembly approved the Global Vaccine Action Plan (GVAP) as the framework for achieving the “Decade of Vaccines” vision of delivering universal access to immunization (8). In 2015, the WHO Strategic Advisory Group of Experts (SAGE) on Immunization emphasized the importance of a MNI platform, calling upon the Organization to affirm its commitment to building evidence for vaccine delivery during pregnancy and promoting its potential for prevention among high-risk groups worldwide.

The GVAP was adapted to fit the context of the Region of the Americas. This Regional Immunization Action Plan (RIAP) lays out a roadmap for 2016 – 2020 (9). Its objectives include two indicators to measure progress toward the MDG-5 target of reducing maternal mortality by 75% by 2015: (a) the number of countries that include vaccination against influenza and/or tetanus-diphtheria-pertussis (Tdap; as tracers of maternal vaccination) in their national immunization schedules; and (b) the number of countries with a hepatitis B birth-dose policy in place.

The Region of the Americas has been a leader in MNI, with years of programmatic experience in vaccinating pregnant women against tetanus, diphtheria, and influenza (10 – 12). In April 2015, the Region of the Americas was declared free of rubella endemic transmission and congenital rubella syndrome, diseases that had affected nearly 20 000 infants annually (13, 14). This paper aims to describe the status of MNI in the Americas, identifying evidence gaps and obstacles to its optimal implementation and discussing opportunities for improvements and the future this crucial, high-impact, public health platform.

## REGIONAL RECOMMENDATIONS

In its recent publication, the *Maternal and Neonatal Immunization Field Guide for Latin America and the Caribbean*, the Pan American Health Organization (PAHO) compiled all the MNI schedules in use by the countries in Latin American and the Caribbean and proposed a common regional schedule, one based on its Technical Advisory Group (TAG) and SAGE recommendations (15). As shown in Table 1, a total of 11 vaccines are considered for use in pregnant women (either routinely or in special situations) and two in newborns. It is indeed imperative that the first doses of hepatitis B and BCG be administered to the newborn within the first 24 hours post-partum.

Countries may engage various complementary approaches to vaccinating pregnant women, depending on the type of vaccine and its availability, the seasonality of the disease, and the immunization strategies in place for different target populations. Vaccine delivery is done through health services depending on the health system/model of the country. Countries of Latin America and the Caribbean typically implement vaccination through prenatal care services, health centers and outreach activities, routine immunization, and mass vaccination campaigns. To ensure success and acceptance of maternal and neonatal immunization, it is important to involve scientific societies and national champions, and to count on well-designed social communication messages.

Health authorities can also encourage vaccination by disseminating information to communities, through midwives and obstetrics-gynecology services. Certain social programs and other activities have also referred pregnant women to health services for vaccination.

### Current state and opportunities for improvement

*Influenza vaccines*. As of 2015, a total of 31 countries and territories in the Americas targeted pregnant women for influenza vaccination. The high burden of disease observed among pregnant women during the 2009 influenza A (H1N1) pandemic contributed substantially to the significant increase in countries targeting this subpopulation, from 7 countries in 2008 to 31 in 2009 (16), as re-emphasized by SAGE in 2012 (17). Information reported by 19 countries of the Americas that target pregnant women for vaccination suggests that the median coverage for this group was 55% in 2015, with significant variation from country to country (16).

Because the influenza vaccine can be given at any stage of pregnancy, there are many opportunities to vaccinate during prenatal care. Regional recommendations emphasize the importance of considering the disease’s seasonality—being sure to vaccinate prior to epidemic peaks in tropical countries and the winter season in temperate countries (18). Nevertheless, in areas of limited access to health services, it may be preferable to vaccinate at the first (and possibly only) prenatal care visit, or at any other medical consultation during pregnancy, with the influenza vaccine formulation available at the time.

*Hepatitis B vaccine*. Most countries in the Americas have included routine hepatitis B vaccination in their national immunization schedules for more than 20 years. This implies that young mothers are already protected against hepatitis B. Some countries, such as Argentina and Brazil, have universal vaccination for the entire population. However, unvaccinated pregnant women who are identified as being at risk for hepatitis B should be vaccinated during prenatal care.

Regarding neonatal immunization against hepatitis B, in October 2016 SAGE re-emphasized its 2009 rec-ommendation that all infants receive a first dose of hepatitis B vaccine preferably within 24 hours of birth (7, 18). The birth dose can still be effective in preventing perinatal transmission if given within 7 days, particularly within the first 3 days, but with declining effectiveness each day. Nonetheless, even after 7 days, a late birth dose can prevent horizontal transmission, and therefore remains beneficial (19). Thus, SAGE recommends that all infants receive the birth dose during the first contact with health facilities at any time before the first primary dose (18). As of October 2016, 35 (69%) of 51 countries/territories in the Americas had included the hepatitis B vaccine birth-dose in their immunization schedules—21 of these for all infants, and 14 for just infants born to hepatitis B virus surface antigen-positive mothers (20, personal communication with national EPI managers). While enough countries have introduced the hepatitis B birth dose to cover 90% of the birth cohort in the Region of the Americas, the timeliness of its administration and its availability have yet to be monitored and guaranteed (20).

**TABLE 1.** Vaccines and their recommended usage and timing in maternal and neonatal immunization programs, per recommendations of the Pan American Health Organization’s Technical Advisory Group (PAHO TAG), 2017

Timing and vaccine type	Pre-pregnancy	Pregnancy	Post-partum	Year of PAHO TAG recommendation
Recommended during pregnancy				
Tetanus	Yes, ideal time.	Yes, two doses if not previously vaccinated.	Yes, to complete schedule.	2017
Inactivated influenza		Yes, ideal time.	Yes, if not vaccinated during pregnancy, to protect tde newborn.	2012
In special situations only				
Tetanus-diphtderia-pertussis		Yes, during outbreaks (ideally from 27 - 36 weeks of gestation).	Yes	2014
Hepatitis B		Yes, IF incomplete schedule and IF under high-risk situation (e.g., more tdan one sexual partner during tde previous 6 montds, sexually transmitted disease, etc.)	Yes, to complete schedule (tdree doses).	NA
Hepatitis A		Yes, during outbreaks.		2013
Yellow fever	Yes, ideal moment (in endemic areas).	Yes, prior to travel to endemic areas under current outbreak, witd prior risk/benefit analysis.		2013
Inactivated polio vaccine		Yes, prior to travel to endemic areas under current outbreak.		2013
Oral polio vaccine		Yes, prior to travel to endemic areas under current outbreak.		2015
Rabies		After high-risk exposure.		2013
Meningococcus conjugate		Yes, during outbreaks		2013
Meningococcus polysaccharide		Yes, during outbreaks		2013
Contraindicated during pregnancy Rubella				
Rubella	Yes, ideal time, but avoid conception for 4 weeks.	No.	Yes, if not vaccinated during pre-pregnancy.	2013
Measles				2013
Mumps				2013
Human papilloma virus	Yes, ideal time.	No.		2013
Recommended for tde newborn				
Bacillus Calmette-Guérin vaccine			As soon as possible after birtd.	2004
Hepatitis B vaccine			Before tde first 24 hours after birtd.	2011

***Source:*** Prepared by the authors from the study data.

The set target of the elimination of hepatitis B mother-to-child transmission by 2020 is expected to boost hepatitis B vaccination by prioritizing it on the national health agendas. Immunization programs should be integrated with maternal and neonatal services, and should facilitate training on vaccine handling, administration, and reporting of the birth-dose vaccination. Health services might consider organizing outreach activities that offer the vaccine for newborns of women giving birth at home. Birth-dose coverage should be recorded separately for doses administered with the first 24 hours and for those administered later. This differentiation in recordkeeping would help detect obstacles to timely vaccination. Current practices and information systems should be adapted for that purpose. The delivery of hepatitis B vaccine within 24 hours of birth should be, in fact, a performance indicator for all immunization programs.

*Tetanus vaccine*. With the exception of Haiti, all countries and territories of the Americas have reached the neonatal tetanus (NNT) elimination goal. Haiti has advanced substantially towards NNT elimination and is augmenting its vaccination activities to achieve this goal. In addition to vaccinating pregnant women during routine immunization activities, in 2013, 2014, and 2015 three rounds of Td vaccination campaigns were conducted in the country’s 140 communes to immunize all women of reproductive age, regardless of previous vaccination status, (personal communication with national EPI manager). Haiti also integrated NNT surveillance into acute flaccid paralysis, measles/rubella, diphtheria, and pertussis case-based surveillance in 2013 (21).

*Pertussis/diphtheria vaccines*. In line with the PAHO TAG recommendations, some of the Region’s countries are administering Tdap to pregnant women as part of campaigns responding to outbreaks (22). However, other countries are giving Tdap routinely to women during every pregnancy. These countries—Argentina, Bahamas, Bermuda, Brazil, Cayman Islands, Colombia, Costa Rica, El Salvador, Mexico, Panama, Paraguay, and Uruguay—should conduct impact evaluations of universal vaccination in order to build local evidence that can inform decisionmaking for other countries in the Region.

## OTHER CONSIDERATIONS FOR SUCCESS

### Integration of programs and services

Achieving the health-related Millennium Development Goals and the next wave of targets beyond 2015 will depend largely on how countries succeed in moving towards universal health coverage. The integration of immunization and other health services delivery has the potential to be mutually beneficial, including improving coverage, saving costs, and creating synergies. However, for integrated efforts to be successful, health system planning and careful forethought is critical. Integrated approaches also need to be supported by sufficient human resources and delivery systems.

Currently, the main obstacles to MNI are insufficient evidence on safety and efficacy of maternal vaccines and a limited knowledge of MNI benefits among obstetricians (compared to pediatricians), resulting in no active promotion of vaccination (PAHO unpublished survey 2015). Insufficient immunization training for resident obstetricians/gynecologists, a lack of active promotion of maternal vaccination at the policy level, and low acceptance of vaccination among health care workers are all important threats to MNI. Inadequate risk communication leads to misconceptions among patients and health care providers. In the case of influenza vaccines, national immunization managers asserted that the language on package inserts, including precautions regarding pregnant women, were sometimes interpreted by medical staff as cautions against use in pregnancy (PAHO Revolving Fund for Vaccine Procurement, unpublished data). There is a need to strengthen coordination of the EPI with existing maternal health programs, health promotion, and communication departments at all levels. The technical guidelines of prenatal health services should include vaccines recommended for pregnant women, and obstetricians/gynecologists and other prenatal health workers should also be appropriately educated and trained.

### Vaccine safety and regulation

In 2014, the WHO Global Advisory Committee on Vaccine Safety (GACVS) conducted a comprehensive review of the evidence on safety of vaccination during pregnancy (23). Reviewing data on various non-live vaccines, including inactivated virus, inactivated bacteria, and the acellular vaccines and toxoids, revealed no safety issues, and GACVS concluded that pregnancy should not preclude women from vaccination when it is indicated (23). Following this review, WHO underscored that the labelling of maternal vaccines should align with existing recommendations and should not use cautionary language where unjustified. Policy regarding use of vaccines in pregnancy is often guided by post-marketing vaccine surveillance systems, as well as data from the small numbers of pregnant women inadvertently vaccinated in clinical trials and campaigns.

Because generating local and regional evidence on vaccines licensed for use during pregnancy is important, countries of the Americas have monitored vaccine safety through surveillance of events supposedly attributable to vaccination or immunization, post marketing surveillance, and safety monitoring conducted by national regulatory authorities. The Latin American Center for Perinatology (CLAP) was established in 1970 with the aim of strengthening health care services with a focus on primary care, and particularly, the health care of mothers and newborns. In recent years, CLAP has compiled data from 29 countries in Latin America through an electronic, standardized perinatology clinical record. This tool has facilitated analyses of maternal and neonatal health outcomes associated with MNI. The CLAP is also establishing a surveillance network of sentinel hospital sites across the Region that will actively look for and investigate suspected cases of potentially MNI-related outcomes in mothers and newborns (24).

### Information systems

The availability of robust and reliable information regarding maternal and child health and maternal and neonatal vaccination is crucial for vaccine introduction, monitoring of program roll-out, vaccine impact evaluation, and monitoring of vaccine safety post-introduction. In recent decades, countries of the Americas have significantly invested in vaccinepreventable disease surveillance, such as sentinel influenza surveillance. However, pregnancy status is not always collected by surveillance forms. Countries systematically report vaccination status for maternal and neonatal immunization to PAHO using the official joint WHO/UNICEF reporting form. Additionally, the clinical data collected through the CLAP information system includes valuable information on prenatal care through delivery, and has recently incorporated variables for vaccination history on its generic regional electronic form. Finally, specific maternal and neonatal indicators should be established and included in the management and oversight of routine immunization programs (Box 1).

#### Social communication and vaccine acceptance

National and international studies have demonstrated that vaccination is less likely in pregnant women with lower socioeconomic status, less educational attainment, and in women belonging to racial/ethnic minority groups (25). Before preparing any informational material, the target population’s knowledge and perception of a disease should be evaluated. This context will permit communicators to determine the information and educational needs of the community and to prepare appropriate content.

BOX 1. **National and Regional indicators relevant to maternal and neonatal immunization, 2017 National indicators**

**National indicators**

- Percentage of pregnant women with at least four antenatal care (ANC) visits
- Percentage of pregnant women with an ANC visit in the first trimester
- Vaccination coverage in pregnant women (Td, influenza, pertussis, hepatitis B)
- Vaccination coverage with the birth dose of hepatitis B within the first 24 hours of life
- Bacillus Calmette-Guérin (BCG) vaccination coverage
- Percentage of AEFI reported with final classification
- Comparability of newborn BCG and hepatitis B vaccination
- Influenza and hepatitis B vaccination coverage among healthcare workers, as a proxy for vaccine acceptance
- among healthcare workers and hence their recommendation of vaccination to the target population

**Regional indicators**

- Number of countries and territories whose immunization schedules include vaccination of pregnant women using influenza and/or tetanusdiphtheria vaccines, as tracers of maternal vaccination
- Number of countries and territories that administer hepatitis B vaccine to newborns during the first 24 hours post-partum
- Number of countries and territories that offer other preventive interventions integrated with vaccination (e.g., prenatal care visits, routine childhood medical visits, iron supplementation, health education, and others)

***Source:*** Prepared by the authors from the study data.

Women’s health providers should explicitly address vaccination with all obstetric patients. Verbal, face-toface recommendations from a physician appear to be a powerful motivator for vaccine acceptance among preg-nant women because they generally view their doctor as a trusted source of medical information. Obstetricians should frame infectious disease prevention for women and infants as a routine part of obstetric care, presenting vaccines as a standard part of anticipatory guidance at first obstetric visits.

### FUTURE OF MATERNAL AND NEONATAL IMMUNIZATION

**Figure 1.**
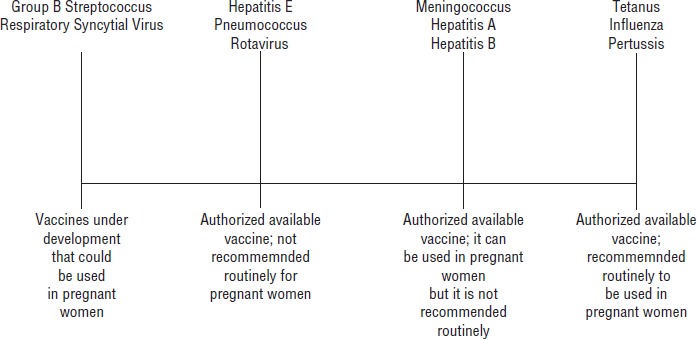
Upcoming vaccines in the development pipeline that are relevant to maternal and neonatal immunization, 2017

There have been important vaccine advances relevant to MNI, including safety and efficacy trials in pregnant women for influenza; clinical trials in pregnancy to support indication of Group B Streptococcus and respiratory syncytial virus; capacity building for clinical pregnancy trials in developing countries; and consensus building on clinical trials studies in pregnancy. Figure 1 shows the upcoming vaccines in the development pipeline that are relevant to MNI.

MNI is expected to be prioritized in the global health agenda in the coming years. Strengthening the MNI platform, in addition to reducing disease burden, is an opportunity to improve the quality, access to, and equity of maternal and neonatal health services in Latin America and the Caribbean. To achieve this goal, it will be critical to strengthen collaborative efforts among agencies, universities, institutions, and other MNI partners, including obstetricians and gynecologists’ societies. Alliance with the media and scientific societies are also imperative for reaching a wide audience, increasing vaccine acceptance and uptake in pregnant women, and actively promoting the integration of immunization with other maternal health services. Moreover, countries should invest in efficient and reliable information registries and strengthen surveillance of events supposedly attributable to vaccination or immunization, including those related to maternal vaccination (e.g., through sentinel hospitals). Building the evidence base to support and inform decisionmaking around MNI should be given priority, and National Immunization Technical Advisory Groups and relevant scientific societies should take part in disseminating MNI recommendations. Countries should also monitor and evaluate the progress and impact of maternal and neonatal immunization.

Given the momentum that MNI is gaining globally and regionally, it is paramount to identify its main strengths, weaknesses, and gaps, as well as any opportunities to strengthen both MNI and other existing programs through synergies and inter-institutional collaboration. Experience from Latin America and the Caribbean indicates that the principal strengths of MNI have been the area’s long-standing tradition of immunization and the trust garnered by it national immunization programs. The history of Td vaccination among pregnant women and the experience of vaccination against influenza-A (H1N1) in pregnant women have further developed the maternal immunization platform in several countries of the Region (11, 12). Political commitment and the prioritization of maternal health on national agendas have also been key to working towards the MDG-5 target and goals for elimination of several vaccine-preventable diseases. Better access to prenatal care and the high proportion of deliveries taking place in facilities (over 92%) have also bolstered vaccination of pregnant women and timely administration of newborn vaccines (20, PAHO/WHO unpublished data).

#### Conclusions

The maternal and neonatal immunization platform is a very promising approach for reducing morbidity and mortality associated with vaccine-preventable diseases among this important population group. Building on significant programmatic experience with vaccination, the countries of the Americas have successfully introduced and sustained maternal and neonatal vaccines—Td, Tdap, and influenza for pregnant women, and BCG and hepatitis B for newborns (10, 11). These introductions have been facilitated by various factors, including alliances with relevant scientific societies, the population’s trust in national immunization programs, and effective social communication.

There is a growing body of research evidence at the global level on the potential benefits of maternal immunization and safety of maternal vaccines. There is also evidence for potential new vaccines for mother and child, such as Group B Streptococcus and Respiratory Syncytial Virus vaccines. Nevertheless, the Region of the Americas, particularly Latin American and the Caribbean, would benefit from generating more local evidence on vaccine effectiveness and safety among pregnant women to support decisionmaking on MNI. This is particularly true for influenza vaccination, which is more widely used in this region than in others; and for Tdap vaccination, which 15 countries have introduced into their universal routine immunization schedules. In addition, the recent creation of the CLAP hospital network offers a unique opportunity to address research needs through routine monitoring of the safety and effectiveness of maternal immunization and to evaluate MNI impact.

This review of the current status of MNI suggests that challenges persist in providing universal and equitable access to MNI. In order to reach more pregnant women, vaccination acceptance among health care workers and their willingness to recommend it to the target population are crucial for increasing maternal vaccine uptake. Comprehensive efforts are needed to maximize the obstetric provider’s recommendation and administration of all MNI. Thus, technical guidelines for prenatal health services should include information on vaccines for pregnant women. Other prenatal health workers should be appropriately educated and trained on the technical and communication aspects of MNI. Developing further alliances with the media and obtaining endorsements from recognized obstetric/gynecological societies will be important for raising vaccine acceptance and uptake in pregnant women.

The integration of immunization with other health services has the potential to be mutually beneficial by improving coverage, saving cost, and creating synergies that advance all toward universal health coverage. However, for integrated efforts to be successful, health system planning and careful forethought are critical. Integrated approaches also need to be supported by sufficient human resources and adequate delivery systems. In order to avoid straining a weak or fragile health system, integrated interventions should be carefully selected, and then monitored to identify any unforeseen challenges and correct them in a timely way. Finally, the strengthening of the MNI platform may facilitate the introduction of upcoming vaccines, while generating the evidence needed by vaccination programs to make decisions and sustain introductions in the future.

#### Acknowledgements.

The authors would like to thank Saad Omer, the William H. Foege Professor of Global Health and professor of global health, epidemiology, and pediatrics at Emory University (Atlanta, Georgia, United States); the schools of public health and medicine in Latin America and the Caribbean; and the Latin American Center of Perinatology for their contributions to PAHO efforts in maternal and neonatal immunization in the Region of the Americas.

#### Disclaimer.

Authors hold sole responsibility for the views expressed in the manuscript, which may not necessarily reflect the opinion or policy of the *RPSP/PAJPH* and/or PAHO.
